# Feasibility of Physical Activity Assessment with Wearable Devices in Children Aged 4–10 Years—A Pilot Study

**DOI:** 10.3389/fped.2018.00005

**Published:** 2018-01-26

**Authors:** Jan Müller, Anna-Maria Hoch, Vanessa Zoller, Renate Oberhoffer

**Affiliations:** ^1^Faculty of Sport and Health Sciences, Institute of Preventive Pediatrics, Technical University of Munich, Munich, Germany

**Keywords:** daily activity, wearable, Garmin vivo jr, applicability, feasibility

## Abstract

**Objective:**

Physical activity (PA) is associated with multiple beneficial health outcomes. Unfortunately, current studies report an alarming decrease of PA throughout all age groups. This study aims to assess general feasibility and PA levels of kindergarten and primary school children with wearable technology specifically manufactured for young children.

**Patients and methods:**

From April 2017 to August 2017, a total of 59 children (7.1 ± 1.7 years, 34 girls) recorded their PA for seven consecutive day wearing a wearable bracelet (Garmin vivofit jr). Afterward, they filled out a short, child-oriented questionnaire to rate the feasibility.

**Results:**

The general feasibility of the devices was rated as rather well regarding size, materials, and wearing comfort. Moreover, children achieved a mean of 83 ± 18 min of moderate-to-vigorous physical activity (MVPA) and 12.202 ± 2.675 steps per day on a weekly average. Therefore, 52 (88.1%) children, and almost all boys (96%), fulfilled the WHO criteria of 60 min of MVPA per day on a weekly average.

**Conclusion:**

Wearables bracelets seem to be feasible devices for PA assessment even in young children. Nevertheless, their potential to increase PA for primary and secondary prevention of cardiovascular disease, as well as the long-term compliance needs to be clarified in further studies.

## Introduction

Being physically active is one of the most important cornerstones for people of all ages to maintain physical and mental health (1). Lack of physical activity (PA) is associated with multiple of non-communicable diseases (NCD) and was therefore named the fourth leading risk factor of NCD by the WHO in 2009 (2). Since the first PA recommendations for adults were published in 1995 from the Center for Disease Control (CDC) (3), several modifications have been issued and specific recommendations addressed, in particular for children and elderly people (4, 5). In addition, many countries have established national recommendations and guidelines to increase PA throughout the population. This was mainly caused by a lot of sectional and epidemiological studies reporting continuously decreasing PA in adults, adolescents, and children (6). Unfortunately, measuring PA is challenging and methodologies range from labor intensive direct observation, over secondary measures of heart rate monitoring or accelerometry, to subjective measures of self-report (7).

In light of these different approaches, studies showed that recalled PA in children is difficult and flawed (8, 9). More than that, accelerometer-based devices that are worn around the hip appear to be too technical and are described as awkward to wear. However, over the past few years more and more consumer appealing commercial wearable activity trackers (e.g., Fitbit, Jawbone, TomTom, Garmin) for adults have entered the marked, also expanding opportunities to integrate such new technology into research. In 2016, Garmin has pioneered with the vívofit^®^ jr as the first wearable bracelet just for children below the age of 10. With controversial reactions in media forums, some praised the technical innovation with its potential benefits of encouraging the very young to more PA. However, others argued it is simply a commercial overkill and a new panoptical tool for helicopter parents. As some of these arguments are understandable, the potential for early childhood prevention and opportunity to fundamentally shape children’s relationship with PA cannot be overlooked (10).

The aim of this pilot study is to assess general feasibility and PA levels in children aged 4–10 years with a wearable bracelet specifically manufactured for this age group.

## Patients and Methods

### Study Subjects

From April 2017 to August 2017, a convenience sample of 59 healthy children (7.1 ± 1.7 years, 34 girls) participated in a wearable-based PA assessment to estimate the feasibility of the devices. Participants were therefore recruited in several Kindergartens and after-school care centers in Bavaria and Baden-Wüerttemberg to voluntary participate in a 1-week trial. Weight and height were assessed in the morning and body mass index (BMI) calculated by weight in kilograms divided by the square of the height in meters. BMI values were transformed into *z*-scores according to German reference values from Kromeyer-Hauschild et al. (11).

The study was designed in accordance with the declaration of Helsinki (revision 2008) and approved by the local ethical board of the Technical University of Munich (project number: 314/14). All children were verbally informed about the meaning and purpose of the study and agreed to participate. Written informed consent was obtained from all guardians.

### Physical Activity Assessment

The Garmin vivofit^®^ jr is a wearable bracelet designed specifically for children from 4 to 9 years of age to track PA in terms of steps and moderate-to-vigorous physical activity (MVPA) in minutes per day. According to the manufacturer, the children-friendly design is comfortable, durable, and waterproof. The wearable had to be paired with a mobile phone for the parents and children to interact with the device and control the settings. In addition, the app offers an interactive gamification concept in which children can earn coins to redeem for agreed-upon rewards managed by the parents. The device has shown to be accurate in assessing PA and steps (12–14) but not energy expenditure (13, 15).

Children and their guardians were instructed with a standardized information sheet on how to pair the bracelet with an app on their mobile phone. They were also instructed to wear the bracelet on seven consecutive days even during leisure time and school sport. The only time the watch could be removed was overnight.

For statistical purposes, MVPA minutes and steps for every day were analyzed and also computed to a weekly average.

### Assessment of Feasibility

Feasibility was assessed after the 7-day trial with a short questionnaire in which children had to answer five questions regarding the convenience of the bracelet. In detail, the questions were as follows: “Did you like to wear the watch?” “Was the watch comfortable for you?” “How have you tolerated the material of the watch?” “How do you like the size of the watch?” and “Has the watch motivated you to be more active?” Children responded on a 5-point pictorial (smiley) Likert scale that was later transformed in a scale from “−2,” “−1,” “0,” “+1,” “+2” with “0” as neutral anchor.

### Data Analyses

Data are described by mean ± SD for all variables.

All analyses were performed using SPSS 23.0 software (IBM Inc., Armonk, NY, USA).

## Results

The feasibility of the wearable was rated rather well from the children in regard to size, materials, and wearing comfort (Table 1).

**Table 1:** Study subjects.

Anthropometrics	
Sex (girls)	34 (57.6%)
Age (years)	7.1 ± 1.7
Body height (cm)	125.8 ± 10.8
Body weight (kg)	26.5 ± 7.8
Body mass index (*z*-score)	0.09 ± 0.91

**School type**	
Kindergarten	35 (59.3%)
Primary school	18 (30.5%)
All-day school	6 (10.2%)

**Feasibility**	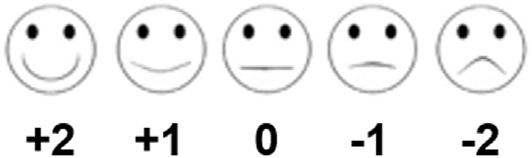

Did you like to wear the watch?	1.8 ± 0.4
Was the watch comfortable for you?	1.0 ± 1.2
How have you tolerated the material of the watch?	1.8 ± 0.4
How do you find the size of the watch?	1.3 ± 0.8
Has the watch motivated you to be more active?	0.6 ± 1.2

**Figure 1:**
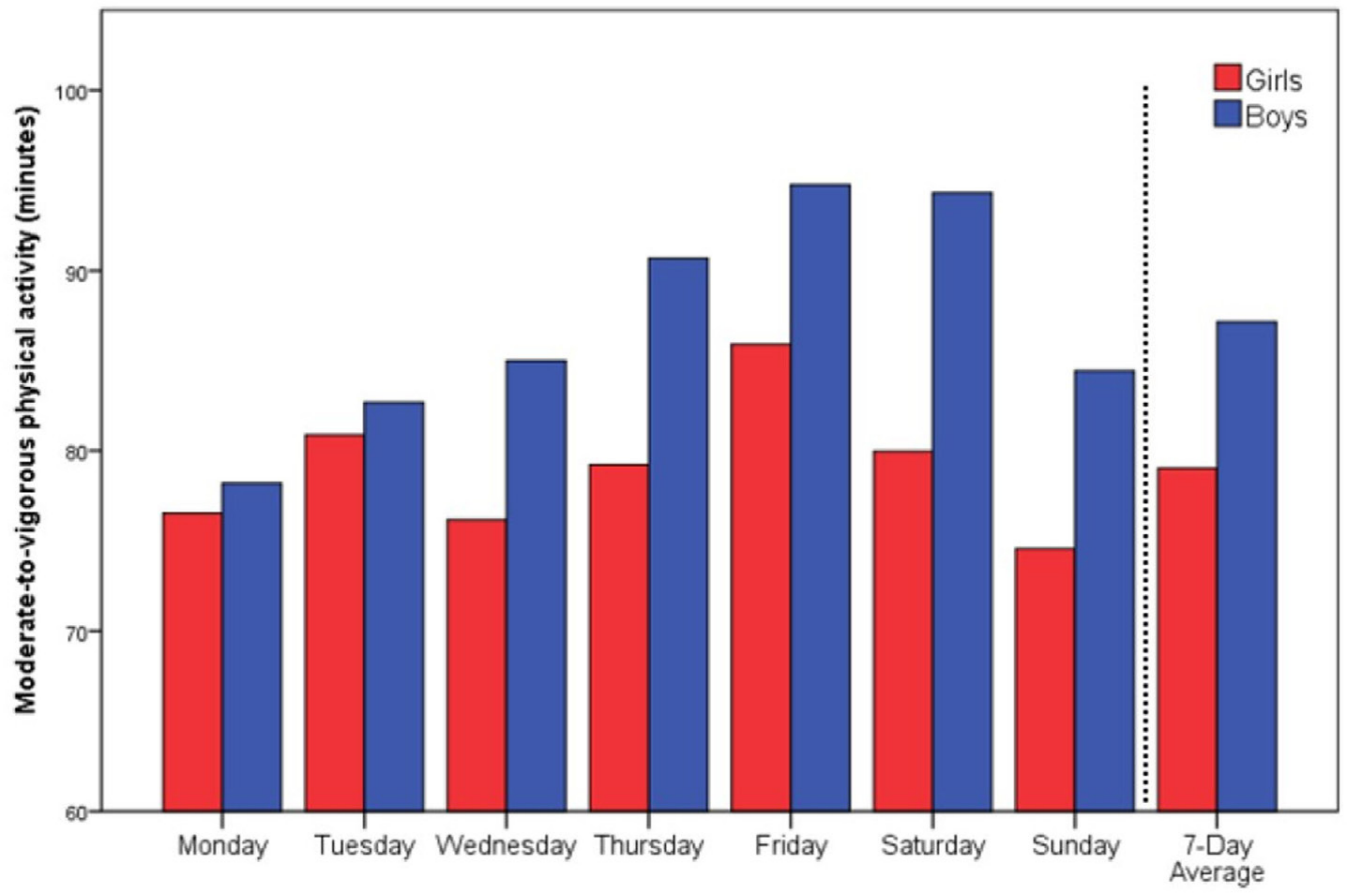
Daily moderate-to-vigorous physical activity minutes according to weekdays.

Children achieved a mean of 83 ± 18 min of MVPA and a total 12.202 ± 2.675 steps on a weekly average of 7 days. In addition, 52 (88.1%) children, and almost all boys (96%), fulfilled the WHO criteria of 60 min of MVPA per day on a weekly average. There was a slight incline in MVPA from Monday to Friday that was more present in boys (Figure 1).

## Discussion

This study outlined that the feasibility of the wearable was rather well in regard to size, materials, and wearing comfort. Moreover, the majority of children and almost all boys fulfilled the WHO (5) criteria of 60 min of MVPA per day on a weekly average and 31 children (53.4%) reached at least 60 min of MVPA on every day of the week.

Assessing PA levels in children is challenging. Previous studies point out that subjective measured PA levels recalled in questionnaires or activity logs showed only a weak correlation to measured PA by accelerometry (8). However, in big cohort studies self-report is still the only applicable method, whereas accelerometry is the method of choice in research projects with a smaller sample size (16). The latter is currently also the gold standard of objective measurement when taking feasibility, applicability, and salary in different settings into account (8, 16, 17).

Nowadays, wearable technology is also more and more used to assess PA since an appealing design and consumer friendly usage have led the line between consumer health wearables and medical devices begin to blur (10, 18). Our pilot study outlines now that the feasibility and acceptance of those devices is very good during a 1-week trial. Especially, the fact that children reported that they liked to wear the watch and that they had no problems with the materials is promising for the long-term use. Initial interest at recruitment and compliance with wear time protocols is much improved for wrist-based commercial activity trackers, as compared with the less-appealing research-grade accelerometer worn with a belt around the hip (19). The appealing design and accessory character are also crucial factors for long-term compliance, which in fact is an aspect that needs to be proven in further studies. That inconspicuous design is also beneficial when it comes to medical application of those wearables, because wearing such a device does not evoke inconvenient questions for the patients. Even in older adults the commercially available wearable appeared to be useful and acceptable (20). Regarding PA assessment, there is just one major concern in general. Wearing such a device for the first time leads to a higher activity in the first days or week of wearing which then declines to normal PA levels over time (21).

In young children, however, this is certainly different. Seven-year-old children are not able to understand the rational of PA for health outcomes, nor to interpret the PA measures on the watch at all. Therefore, it is unlikely that they are more active just because of the bracelet. This assumption is even reflected in the neutral response to the question in our questionnaire: “Has the watch motivated you to be more active?” On the other hand, fashion concerns were already present in this young age group which might be a reason why the feasibility of this children-friendly designed wearable turned out to be good to very good in regard to materials, comfort, and size and why the children reported they liked to wear the bracelet very much.

Regarding the technical applicability, studies have shown that wearable bracelets or watches track steps and activity minutes at the wrist as accurate as accelerometers (12–14), although technological details and algorithms of the manufactures are kept confidential. However, it should be mentioned that agreement between devices from different manufacturers is generally poor. This is due to different interpretation of guidelines and the use of different cut points (thresholds) for MVPA. This ambiguity resulting in a span from almost 0 to 95% in preschool children complying with PA recommendations (22) makes generalization impossible which is why it is recommended that estimates are usable only within their own cohort of measurement (4, 16).

## Conclusion

Overall, wearable bracelets seem to be feasible devices for PA assessment even in young children. The appealing design and the relatively low price in comparison to triaxial or heart-rate-based accelerometers make them applicable in broader cohort studies. Nevertheless, their potential to increase PA for primary and secondary prevention of cardiovascular disease needs to be clarified in further studies.

### Study Limitations

Several limitations are reported with the use of pictorial Likert scales in children (23, 24) and a possible scoring bias in feasibility could not entirely be ruled out. Moreover, the questionnaire lacks standardization and the voluntary recruitment of participants may have led to an overestimation of PA in this cohort.

Accelerometer-assessed PA is biased due to different devices and utilization of different cutoff points to determine MVPA when using accelerometry (25–28). These drawbacks make a comparison of our data possible only within our own cohort.

## Ethics Statement

The study was prospectively designed in accordance with the declaration of Helsinki (revision 2008) and approved by the local ethical board of the Technical University of Munich (project number 314/14). All children were orally informed on the meaning and purpose of the study and agreed to participate. Written informed consent was obtained from all guardians.

## Author Contributions

JM was responsible for conception, design of the study, sampled parts of the data, analyzed the data, and drafted the manuscript. A-MH and VZ sampled the data and gave important input for drafting and revising the manuscript. RO was responsible for conception and design of the study and gave important input for revising the manuscript.

## Conflict of Interest Statement

The authors declare that the research was conducted in the absence of any commercial or financial relationships that could be construed as a potential conflict of interest.
